# Inferior Vena Caval Thrombosis After Traumatic Liver Injury

**DOI:** 10.1155/1998/53169

**Published:** 1998-12

**Authors:** Takeo Kimoto, Hitoshi Kohno, Masaaki Uchida, Akira Yamanoi, Akitaka Yamamoto, Naofumi Nagasue, Seiichi Ando, Kouya Suemitsu, Mitsuru Ohtani

**Affiliations:** 1 Second Department of Surgery Shimane Medical University Izumo 693 Japan; 2 Department of Surgery Unnan General Hospital Ohara County Shimane 699-12 Japan; 3 Department of Neurosurgery Unnan General Hospital Ohara County Shimane 699-12 Japan

## Abstract

We report here the case of a 35-year-old man who presented with inferior vena cava thrombosis (IVCT) after blunt hepatic trauma. The IVCT was incidentally detected by computed tomography (CT) 35 days after deep parenchymal suturing and suture approximation for liver lacerations. The patient denied any symptoms of thrombophlebitis. However, he had presented with significantly elevated values of FDP-D-dimer and a modest increase in plasminogen concentration, which indicated that he had been in a hypercoagulable and hypofibrinolytic state after the operation. He had not undergone any prophylactic anticoagulant therapy because of his concomitant subarachnoid hemorrhage and huge hepatic hematoma. The patient was treated with an emercy thrombectomy. Posttraumatic IVCT is extremely rare phenomenon. We should consider IVCT in patients with a severe hepatic injury, particularly if their coagulation system change into hypercoagulable and hypofibrinolytic state. Additionally, this case made us reflect on the treatment of traumatic liver injury.


*HPB Surgery*, 1998, Vol. 11, pp. 111–116
Reprints available directly from the publisher
Photocopying permitted by license only

© 1998 OPA (Overseas Publishers Association) N.V.
Published by license under
the Harwood Academic Publishers imprint,
part of The Gordon and Breach Publishing Group.
Printed in Malaysia.

### *Case Report*

### Inferior Vena Caval Thrombosis after Traumatic Liver Injury

TAKEO KIMOTO[a,*], HITOSHI KOHNO[a], MASAAKI UCHIDA[a], AKIRA YAMANOI[a], AKITAKA YAMAMOTO[a], NAOFUMI NAGASUE[a], SEIICHI ANDO[c], KOUYA SUEMITSU[c] and MITSURU OHTANI[b]

[a] *Second Department of Surgery, Shimane Medical University, Izumo 693, Japan;* [b] *Department of Surgery;* [c] *Department of Neurosurgery, Unnan General Hospital, Ohara County, Shimane 699-12, Japan*

*(Received 20 April 1997)*

**We report here the case of a 35-year-old man who presented with inferior vena cava thrombosis (IVCT) after blunt hepatic trauma. The IVCT was incidentally detected by computed tomography (CT) 35 days after deep parenchymal suturing and suture approximation for liver lacerations. The patient denied any symptoms of thrombophlebitis. However, he had presented with significantly elevated values of FDP-D-dimer and a modest increase in plasminogen concentration, which indicated that he had been in a hypercoagulable and hypofibrinolytic state after the operation. He had not undergone any prophylactic anticoagulant therapy because of his concomitant subarachnoid hemorrhage and huge hepatic hematoma. The patient was treated with an emercy thrombectomy. Posttraumatic IVCT is extremely rare phenomenon. We should consider IVCT in patients with a severe hepatic injury, particularly if their coagulation system change into hypercoagulable and hypofibrinolytic state. Additionally, this case made us reflect on the treatment of traumatic liver injury.**

*Keywords:* Inferior vena cava, thrombosis, trauma, liver, coagulation

### INTRODUCTION

Deep venous thrombosis (DVT) is a significant source of morbidity and mortality in patients with a major traumatic injury. The reported incidence of DVT in patients with trauma varies between 20% and 90%, whereas the incidence of pulmonary embolism is from 4% to 22% [1]. Despite the high incidence of DVT, inferior vena caval thrombosis (IVCT) after blunt trauma is extremely rare. To our knowledge, only ten such cases have been presented in the English literature since 1911 [2–10]. The case reported here was IVCT which was incidentally detected 35 days after deep parenchymal suturing and suture approximation for the traumatic liver lacerations. The patient had suffered from an infected liver hematoma and presented a coagulation disorder before the detection of IVCT.

*Corresponding author.

The mechanism of posttraumatic IVCT and the treatment of hepatic trauma are discussed.

### CASE REPORT

An intoxicated 35-year-old man was taken to the emergency room of Unnan General Hospital after falling from a height of 3 meter. He was semicomatose upon arrival (Glasgow Coma Scale (GCS) score 7). He presented anisocoria and right hemiplegia. Subcutaneous bleeding was found at his occipital and right hypochondrial regions. At first, he was hemodynamically stable. Chest X-ray revealed neither pneumothorax nor hemothorax. Computed tomography (CT) of the head demonstrated suba-rachnoid hemorrhage at the brain surface of the left cerebral hemisphere and in the left ambient and the basal cysterns. A concurrent abdominal CT showed hepatic laceration with a parenchymal hemorrhage in Segment 6 and 7, and small amount of intraabdominal fluid. It showed no retroperitoneal hematoma (Fig. 1).

The patient was admitted to the intensive care unit and treated conservatively because his hemodynamic state was stable. Ten hours later, however, he was transferred to Shimane Medical University for an emergency operation, since the bleeding from the hepatic lacerations was continuing. By this time, eleven units of packed red blood cells had been transfused.

At laparotomy, the hepatic lacerations 5 cm in length were revealed both in segment 6 and 7. Intraabdominal blood accumulation was 4000 ml. Although active bleeding was not found from the hepatic wounds, the passive oozing continued, which was controlled by deep parenchymal suturing and suture approximations for the lacerations.

The postoperative course was initially stable. GCS score of the patient returned to 14 by the second postoperative day (2POD). The CT of the head showed that the subarachnoid hemorrhage in density and range decreased. However, he had complained of mild hypochondriac pain and low grade fever (37.0 – 37.8 degrees). Additionally, leukocytosis persisted (15,000/mm 3 – 2,0000/ mm 3). The abdominal CT on the 7th POD demonstrated the 6 cm. Sized hepatic hematoma in segment 7, which was drained percutaneously. The bacteriological examination revealed that the drained hematoma contained coagulase-negative staphylococcal species (3+). After the drainage and antibiotics, the low grade fever and leukocytosis subsided. He began to walk around the bed side although he occasionally showed a confusion due to the brain contusion. Prophylactic anticoagulant therapy had not been indicated since rebleeding from the hepatic hematoma or the brain

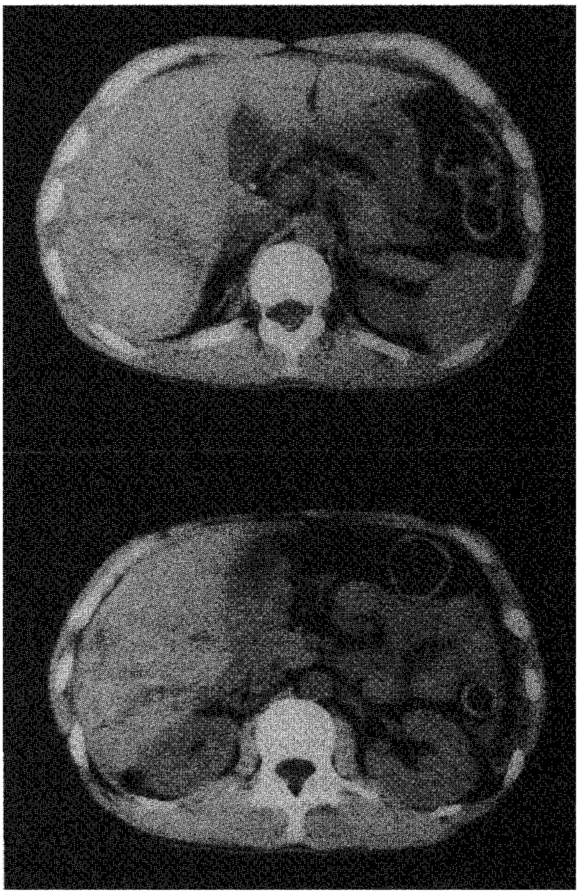

FIGURE 1 CT scan at admission. We can see the hepatic parenchymal hemorrhage extending to the inferior vena cava at the posterior portion of the liver. Retroperitoneal bleeding was not seen.

was a concern. On the 35POD, an abdominal CT for the infected hematoma unexpectedly detected an inferior vena caval thrombus, 4 cm in length, extending caudally from 3 cm below the renal vein (Fig. 2). The patient denied any symptoms of thrombophlebitis of his lower extremities. Laboratory data at that time included: Hct, 32.6%; WBC, 19,600/mm 3; Plt, 404,000/mm3; total bilirubin, 2.0 mg/dl; direct bilirubin, 0.7 mg/dl; AST, 183IU/L; ALT, 101IU/L; ALP, 71IU/L; g-GTP, 29IU/L; LDH, 859IU/L; BUN, 28 mg/dl; creatinine, 1.3 mg/dl; normal electrolytes; prothrombin time, 13.1 seconds; activated partial thromboplastin time, 33.9 seconds; FDP, 7.7 mg/ml; FDP D-dimer, 2.9 mg/ml.

Since the thrombus seemed to be freely floating in the IVC on CT scan (Fig. 2), the patient underwent an emergency vena caval thrombectomy. Actually, the thrombus was only partly fixed to the caval wall and was floating on intraoperative ultrasonography. The insertion of a vena cava filter was not performed since the following thrombolytic therapy might cause rebleeding from the hepatic drainage. Soon after the thrombectomy, the patient became afebrile and leukocytosis returned normal. He was discharged from the hospital 4 months after the operation. He has been doing well for the last 18 months.

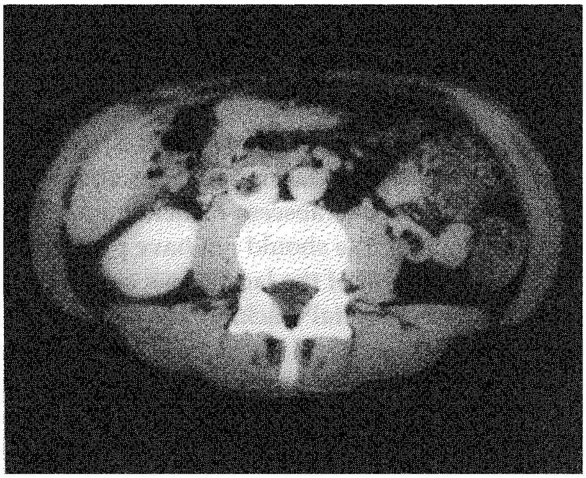

FIGURE 2 CT scan obtained 35 days after injury. Note the soft-tissue density area, 1 cm in width, along the inferior vena cava.

### DISCUSSION

Inferior vena caval thrombosis (IVCT) after blunt trauma is extremely rare. To our knowledge, only ten such cases have been presented in the English literature since 1911 [2–10]. Three different mechanisms of IVCT after blunt trauma have been advocated; 1) caval stasis secondary to compression by a pericaval or retroperitoneal hematoma [2, 4] 2) partial disruption or endothelial injury of the caval wall with secondary thrombus formation [3, 5, 8, 10] and 3) hepatic vein thrombosis after liver trauma extends into the inferior vena cava [9]. In our case, the second explanation is tenable, since the CT scan revealed neither retroperitoneal hematoma around the IVC nor thrombus in the hepatic vein. However, it showed a huge hepatic parenchymal hemorrhage extending to the retrohepatic portion of the IVC, and we supposed the IVC to be bruised (Fig. 1).

Hypercoagulability associated with suppression of fibrinolysis is a normal physiologic response after trauma [11]. Enderson *et al.*, described D-dimer cross-linked fibrin degradation product (FDP-D-dimer) in trauma patients as an indicator of hypercoagulability [12]. They advocated that a second rise in FDP-D-dimer following the initial increase correlated strongly with the development of venous thromboembolism [13]. Alterations in the fibrinolytic system are concurrent with this hypercoagulability. After the initial hyperfibrinolytic state, overall fibrinolytic activity is strikingly reduced. Figure 3 shows the time course of changes in the values of hypercoagulability (FDP-D-dimer, platelet count, and fibrinogen) and fibrinolysis (plasminogen) in our patient. Although plasminogen itself could not be a direct indicator of fibrinolysis, we refer to it as an indirect indicator as Kapsch described [14]. Since the initial

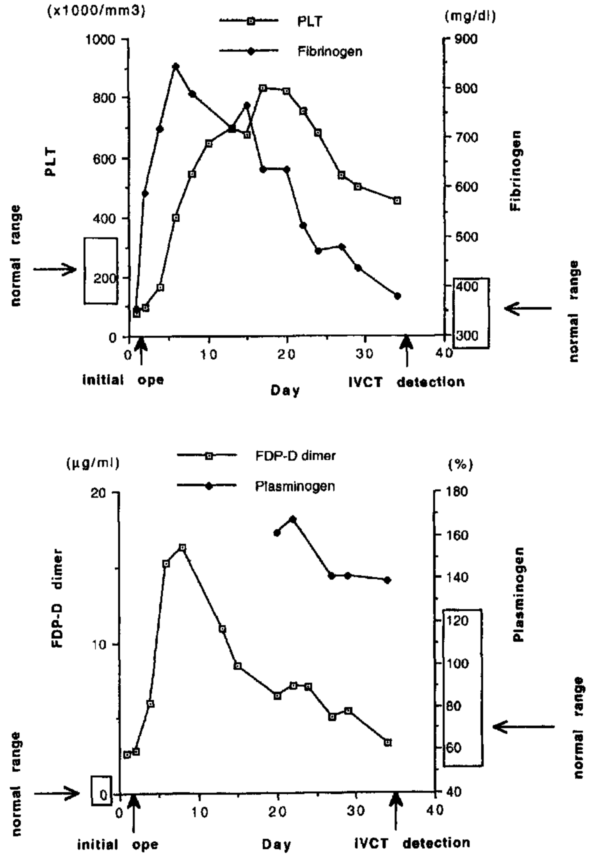

FIGURE 3 Time course of changes in Platelet and Fibrinogen (above), and in FDP-D dimer and Plasminogen (below).

operation, our patient had presented with significantly elevated values of FDP-D-dimer, platelet count, and fibrinogen possibly due to continuing intrahepatic bleeding and associated infection. The modest increase of plasminogen concentration had been also found from one week before the IVCT detection. These phenomena indicated that he had been in the hypercoagulable and probably hypofibrinolytic state. In our opinion, the IVCT in our patient was caused by an endothelial injury of IVC, which progressively formed thrombus with the background of a hypercoagulable and hypofibrinolytic condition.

Based on the review of IVCT after trauma (Tab. I), the incidence of hepatic laceration preceding IVCT was relatively high. Among the eight cases which described the associated injury with IVCT, four patients, including our patient, were reported to have a laceration of the liver [3, 6, 8]. We should consider that such a severe blunt trauma as to cause liver laceration may accompany the endothelial injury of the IVC and increase the risk of IVCT. In view of our experience, we recommend that IVCT be considered in patients with a severe hepatic injury, particularly if their coagulation systems change into a hypercoagulable and hypofibrinolytic state. A series of CT examinations should be mandatory to make an early diagnosis of IVCT.

The current trends appears to swing to a less aggressive approach in the management of liver injury. Recently, the nonoperative management has been applied to hemodynamically stable patients [15]. However, some proportion of the injury require a formidable challenge for major hemorrhage from deep parenchymal fractures or lobar disruption, including packing techniques, resectional debridement, hepatotomy with vascular ligation and major hepatic resection [16]. In our case, we finally chose the surgical intervention (deep parenchymal suturing and suture approximation) because the bleeding from the lacerated liver did not cease. Consequently, this procedure made the postoperative hepatic abscess. Sutures placed tight to compress the bleeding might in fact embarrass the blood supply to the hepatic parenchyma and cause the abscess formation. Levin *et al.*, warned that closing hepatic lacerations promoted the formation of hepatic abscess or hematoma with hematobilia [17]. We should not have performed the approximation of the lacerations and should have elected omental packing or hepatotomy with vascular ligation in the current case. Fabian *et al.*, recommended omental packing as the most effective treatment for most major hepatic injuries [18]. They applied this procedure in 87 patients (60%) of their major hepatic injuries, yielding an abscess rate of 8%. This result was superior to the other treatments, including

TABLE I Summary of posttraumatic IVCT

| Author (Ref) | Age/Sex | Cause of IVCT | Associated injury | Interval between Trauma and Detection | Symptoms /Diagnosis | Treatment /Outcome |
|---|---|---|---|---|---|---|
| Little and Montgomery (1952) (10) | 57/M | Partial disruption of IVC | Right costal fractures Hemothorax | 7 months | Asites Foot edema (Budd-Chiari) /Autopsy | none/Dead 8 months after trauma |
| Hales and Scatliff (1966) (7) | 28/M | – | – | 7 years | Hematemesis Hepatomegaly (Budd-Chiari) /Venography | -/Dead 2 years after trauma |
| Deutsch *et al.* (1972) (9) | – <br> – | Initial IHVT extending IVC | – | – | Hematemesis Hepatomegaly Abdominal pain (Budd-Chiari)/ Venography | -/Dead 22 weeks after Trauma |
| (Two cases) | – | – | – | – | | - |
| Grmoljez *et al.* (1976) (4) | 57/M | Retroperitoneal hematoma | none | 4 months | Back pain, DVT PE, hepatomegaly (Budd-Chiari)/ Operation | Thrombectomy Heparin/Alive |
| Campbell *et al.* (1981) (3) | 21/M | Partial disruption of IVC | Liver laceration | 19 days | Lower abdominal pain Pleural effusion/CT, US | Heparin Coumadin/ Alive |
| Nagy *et al.* (1990) (5) | 55/M | Endothelial Injury | Paraletic ileus | 16 days | Abdominal and low back pain/CT | Greenfield filter Coumarin/Alive |
| Knudson *et al.* (1992) (6) | 33/F | – | Head Injury Splenic and liver laceration femur fracture | 21 days | PE/CT | Heparin Coumadin/- |
| Takeuchi *et al.* (1995) (2) | 21/M | Retroperitoneal hematoma | Pelvic fractures Cerebral contusion | 14 days | Lower abdominal pain, PE/Autopsy | None/Dead 14 days after trauma |
| Patel *et al.* (1996) (8) | 36/F | Endothelial Injury | Liver laceration Fractures of extremities Perforation of small intestine | 5 weeks | DVT, Pleural effusion, Acsites (Budd-Chiari)/ CT | Percutaneous mechanical thrombectomy Heparin, Urokinase/Alive |
| Present case | 35/M | Endothelial Injury | Liver laceration Subaracnoid hemorrhage Hepatic hematoma | 35 days | Right hypochondrial pain/CT | Thrombectomy/ Alive |

IVC: inferior vena cava, DVT: deep venous thrombus, IHVT: intrahepatic venous thrombus, PE: pulmonary embolism, Ref: reference.

gauze packing (an abscess rate of 30%), resectional debridement (15%), and large vessel ligation (14%).

The traditional approach to venous thromboembolism prophylaxis has been the use of subcutaneous heparin or venous compression boot. However, the clear-cut benefit of these treatments in trauma patients has not been presented in the literature [19]. In our case, prophylactic anticoagulant therapy to venous thrombosis was not considered because of hemorrhagic risk. In addition, we did not feel the necessity of venous compression boot since he could ambulate. However, we cannot justify the "no prophylactic treatment" since the patient was at high risk of venous thrombosis because of multiple injuries and hypercoagu-ability. The use of low molecular weight heparin (LMWH) in high-risk trauma patients is currently under investigation [20]. Several studies have demonstrated the efficacy of LMWH in preventing venous thromboembolism with less bleeding complications in orthopedic patients [21, 22]. In such major hepatic and head injury as our case, however, the safety and efficacy of LMWH is unclear. Further studies are mandatory to evaluate the detailed indication or contraindication of LMWH in major hepatic or head trauma in preventing venous or pulmonary thromboembolism.

### References

[1] Shackford, S. R. and Moser, K. M. (1988). Deep venous thrombosis and pulmonary embolism in trauma patients. *J. Intensive Care Med.*, **3**, 87–98.

[2] Takeuchi, M., Maruyama, K., Nakamura, M., Chikusa, H., Yoshida, T., Muneyuki, M. and Nakano, T. (1995). Posttraumatic inferior vena caval thrombosis: Case report and review of the literature. *J. Trauma.*, **39**, 605–608.

[3] Campbell, D. N., Liechty, R. D. and Rutherford, R. B. (1981). Traumatic thrombosis of the inferior vena cava. *J. Trauma.*, **21**, 413–415.

[4] Grmoljez, P. F., Donovan, J. F. and Willma, V. L. (1976). Traumatic inferior vena caval obstruction. *J. Trauma.*, **16**, 746–748.

[5] Nagy, K. K. and Duarte, B. (1990). Post-traumatic inferior vena caval thrombosis: Case report. *J. Trauma.*, **30**, 218–221.

[6] Knudson, M. M., Collins, J. A., Goodman, S. B. and McCrory, D. W. (1992). Thromboembolism following multiple trauma. *J. Trauma.*, **32**, 2–11.

[7] Hales, M. R. and Scatliff, J. H. (1966). Thrombosis of the inferior vena cava and hepatic veins (Budd-Chiari Syndrome). *Ann. Int. Med.*, **65**, 768–781.

[8] Patel, N. H., Bradshaw, B., Meissner, M. H. and Townsend, M. F. (1996). Posttraumatic Budd-Chiari Syndrome treated with thrombolytic therapy and angioplasty. *J. Trauma.*, **40**, 294–298.

[9] Deutsch, V., Rosenthal, T., Adar, R. and Mozes, M. (1972). Budd-Chiari Syndrome: Study of angiographic findings and remarks on etiology. *Am. J. Roentgenol. Rad. Ther. Nucl. Med.*, **116**, 430–439.

[10] Little, R. D. and Montgomery, P. O. (1952). Case report of stenosis of the vena cava with vena caval and hepatic vein thrombosis related trauma. *Ann. Intern. Med.*, **37**, 197–203.

[11] Bennett, B. and Towler, H. M. A. (1985). Haemostatic response to trauma. *Br. Med. Bull.*, **41**, 274–280.

[12] Enderson, B. L., Chen, J. P., Robinson, R. and Maull, K. I. (1991). Fibrinolysis in multisystem trauma patients. *J. Trauma.*, **31**, 1240–1246.

[13] Schmidt, U., Enderson, B. L., Chen, J. P. and Maull, K. I. (1992). D-dimer levels correlate with pathologic thrombosis in trauma patients. *J. Trauma.*, **33**, 312–320.

[14] Kapsch, D. N., Metzler, M., Harringtom, M., Mitchell, F. L. and Silver, D. (1984). Fibrinolytic response to trauma. *Surgery*, **95**, 473–478.

[15] Croce, M. A., Fabian, T. C., Menke, P. G., Waddle-Smith, L., Minard, G., Kudsk, K. A., Patton, J. H., Schurr, M. J. and Pritchard, F. E. (1995). Nonoperative management of blunt hepatic trauma is the treatment of choice for hemadynamically stable patients. *Ann. Surg.*, **221**, 744–755.

[16] Reed, R. L., Merrell, R. C., Meyers, W. C. and Fischer, R. P. (1992). Continuing evolution in the approach to severe liver trauma. *Ann. Surg.*, **216**, 524–538.

[17] Levin, A., Gover, P. and Nance, F. C. (1978). Surgical restraint in the management of hepatic injury: A review of Charity Hospital experience. *J. Trauma.*, **18**, 399–404.

[18] Fabian, T. C., Croce, M. A., Stanford, G. G., Payne, L. W., Mangiante, E. C., Voeller, G. R. and Kudsk, K. A. (1991). Factors affecting morbidity following hepatic trauma. *Ann. Surg.*, **213**, 540–548.

[19] Rogers, F. B. (1995). Venous thromboembolism in trauma patients. *Surg. Clin. North. Am.*, **75**, 279–291.

[20] Knudson, M. M., Morabito, D., Paiement, G. D. and Shackleford, S. (1996). Use of low molecular weight heparin in preventing thromboembolism in trauma patients. *J. Trauma.*, **41**, 446–459.

[21] Green, D., Lee, M. Y., Lim, A. C., Chmiel, J. S., Vetter, M., Pang, T., Chen, D., Fenton, L., Yarkony, C. M. and Meyer, P. R. (1990). Prevention of thromboembolism after spinal cord injury using low-molecular weight heparin. *Ann. Intern. Med.*, **113**, 571–574.

[22] Nurmohamed, M. T., Rosendaal, F. R., Buller, H. R., Dekker, E., Hommes, D. W., Vandenbroucke, J. P. and Briet, E. (1992). Low molecular-weight heparin versus standard heparin in general and orthopedic surgery: *A meta-analysis Lancet.*, **340**, 152–156.

